# The Challenges and Opportunities Associated with Biofortification of Pearl Millet (*Pennisetum glaucum*) with Elevated Levels of Grain Iron and Zinc

**DOI:** 10.3389/fpls.2016.01944

**Published:** 2016-12-23

**Authors:** Hanna R. Manwaring, H. F. J. Bligh, Rattan Yadav

**Affiliations:** ^1^Institute of Biological, Environmental and Rural Sciences, Aberystwyth UniversityAberystwyth, UK; ^2^Unilever Research and DevelopmentSharnbrook, UK

**Keywords:** nutrition, pearl millet, biofortification, plant breeding, genetic improvement

## Abstract

Deficiencies of essential micronutrients such as iron and zinc are the cause of extensive health problems in developing countries. They adversely affect performance, productivity and are a major hindrance to economic development. Since many people who suffer from micronutrient deficiencies are dependent on staple crops to meet their dietary requirements, the development of crop cultivars with increased levels of micronutrients in their edible parts is becoming increasingly recognized as a sustainable solution. This is largely facilitated by genetics and genomic platforms. The cereal crop pearl millet (*Pennisetum glaucum*), is an excellent candidate for genetic improvement due to its ability to thrive in dry, semi-arid regions, where farming conditions are often unfavorable. Not only does pearl millet grow in areas where other crops such as maize and wheat do not survive, it contains naturally high levels of micronutrients, proteins and a myriad of other health benefitting characteristics. This review discusses the current status of iron and zinc deficiencies and reasons why interventions such as fortification, supplementation, and soil management are neither practicable nor affordable in poverty stricken areas. We argue that the most cost effective, sustainable intervention strategy is to biofortify pearl millet with enhanced levels of bioavailable iron and zinc. We discuss how naturally occurring genetic variations present in germplasm collections can be incorporated into elite, micronutrient rich varieties and what platforms are available to drive this research. We also consider the logistics of transgenic methods that could facilitate the improvement of the pearl millet gene pool.

## The Consequences of Micronutrient Deficiencies (Iron and Zinc)

On a global scale, over three billion people suffer from MNDs of essential minerals and vitamins (Chasapis et al., 2012). Deficiencies in iron (Fe) and zinc (Zn) are two of the most common and widespread MNDs (Bailey et al., 2015). This is expensive to society as the consequences include: poor health, increased mortality, low work productivity, learning disabilities in children and poor national economic development at the country level (Welch and Graham, 2004). At an individual level, children require up to 10 mg of Fe and Zn per day (8 mg for adults). This amount is essential to sustain life and to ensure optimal physiological function (Halliwell, 1996; Prasad, 2008; Bailey et al., 2015). Target levels of Fe and Zn required for efficient function of the human body can be seen in **Table 1**.

**Table 1:** Recommended dietary allowances (RDAs) for iron and zinc (Institute of Medicine, Food and Nutrition Board, 2001).

Iron (mg)				Zinc (mg)			
Age	Male	Female	Pregnancy	Age	Male	Female	Pregnancy
0–6 months	0.27	0.27		0–6 months	2	2	
7–12 months	11	11		7–12 months	3	3	
1–3 years	7	7		1–3 years	3	3	
4–8 years	10	10		4–8 years	5	5	
9–13 years	8	8		9–13 years	8	8	
14–18 years	11	15	27	14–18 years	11	9	12
19+ years	8	18	27	19+ years	11	8	11

In developing countries within Africa and Asia, the intake of flesh foods, which are abundant in readily available haem Fe and Zn is at best limited due to economic, cultural, or religious constraints. Instead, staple diets are primarily plant based (Gibson et al., 2000). Non haem Fe and Zn are obtained from a large number of plant based food items such as dark leafy vegetables, brown rice, beans, nuts, and seeds. These are more available generally and are the source of most Fe and Zn in the diet world-wide. People living in poverty stricken areas have limited access to even these foods. Instead, they largely depend on grain from staple crops, which they eat on a daily basis.

Fe deficiency is the most common MND worldwide and can lead to microcytic anemia, impaired immune function and poor endocrine function (Bailey et al., 2015). According to the World Health Organization (2016) over 30% of the global population suffer from anemia due to Fe deficiency; this mostly affects women and children in resource poor areas (Halliwell, 1996). Young children, pregnant and postpartum women are considered to be most at risk due to the high Fe demands associated with infant growth and pregnancy (Stoltzfus et al., 2004). Severe Fe deficiency is associated with increased maternal mortality and can increase the rate of premature delivery and miscarriage (Carriaga et al., 1991). Fe deficiency also contributes substantially to maternal deaths from dystocia (obstructed labor) and is associated with low offspring birth weight (less than 2,500 g). These deficits lead to poor health and low productivity in adulthood as the detrimental effects in childhood persist to adulthood.

Adequate Zn is required for efficient immune function, and deficiency is associated with increased incidence of diarrhoeal diseases and acute respiratory infections, which are major causes of infant mortality (Hambidge, 2000; Black, 2003; Bailey et al., 2015). Zn deficiency is most prevalent in Africa and South-East Asia (Caulfield and Black, 2004) and many studies have demonstrated a relationship between low plasma Zn levels during pregnancy and low birth weight (<5.5l b) within these areas (Neggers et al., 1990; Black et al., 2013). This leads to stunted growth and impaired physical/neural development. Despite the serious health implications, Zn deficiency has received much less attention than other MNDs.

Levels of Fe and Zn deficiency are dependent on a variety of factors, including absorption into the body. Recent studies have proved a direct link between celiac disease and malabsorption of Fe and Zn via the small intestine. The prevalence of celiac disease is estimated to be between 0.5 and 1% in various parts of the world (Gujral et al., 2012), but is especially prominent in grain dependent communities in India and Africa (Green and Cellier, 2007; Rajpoot and Makharia, 2013). The consequences of this disease can snowball into chronic anemia, of which the effects are often irreversible. Celiac disease is precipitated by the consumption of gluten, which is a major storage protein present in barley and wheat (Green and Cellier, 2007). Because of this, doctors usually recommend that therapy is a strictly gluten free diet (Green and Cellier, 2007), which is difficult to achieve in poverty stricken areas. Where lack of food is a problem, to further restrict what a person is able to eat is not feasible. In these circumstances, Pearl millet can be recommended in the treatment of celiac disease because it is naturally gluten free (Nambiar et al., 2011). Pearl millet is sold in a variety of products such as whole raw grains, milled flour, flakes or as a puffed product. The range of processed millet products has also increased and is available in breads, biscuits and pastas (Saturni et al., 2010).

## Drawbacks in Current Strategies to Manage and Prevent Micronutrient Deficiencies

Nutritional dietary supplementation is intended to provide nutrients that may otherwise not be consumed in sufficient quantities through diet alone. Combined Fe and Zn supplementation is perceived to be a logical MND prevention strategy (Lind et al., 2004). Even though studies show significant improvements to human health including weight gain, improved development and a reduction in the incidence of diarrheal disease and lower respiratory infection in children, as measured by the Bayley Scales of Infant Development (BSID), these positive effects are often not experienced without adverse side effects such as vomiting and fever (Lind et al., 2004). For example, Lind et al. (2004) investigated the effects of Fe/Zn supplementation in Indonesian infants over a period of 6 months. Results showed that vomiting as a side effect was observed in 33% of children who took a combined treatment of Fe and Zn, whereas when Fe was taken as an individual supplement, only 18% reported vomiting and 21% reported vomiting for Zn individual supplements (Lind et al., 2004).

The purpose of food fortification is to add essential trace elements and vitamins to food. This aim of which is to “improve the nutritional quality of food and to provide a public health benefit with minimal risk to health” (World Health Organization, Food and Agricultural Organization of the United Nations, 2006). This option also suffers from limiting drawbacks as the fortified compounds at high levels may alter taste, appearance and have a negative effect on shelf life, thus making the product unacceptable to the consumer. For example, the most bioavailable Fe compounds, which are freely water soluble are the most likely to cause unacceptable changes to color and taste (Hurrell, 1997). In some cases, ferrous additions to food may cause a noticeable precipitation reaction, such as when Fe fortified sugar is added to beverages. Many Fe compounds are colored and cause a noticeable change in appearance, especially when added to lighter colored foods. For example, infant cereals have been found to turn gray or green upon the addition of a ferrous sulfate and salt fortified with Fe will turn yellow/brown in color. Negative effects on taste are often reported as a ‘metallic’ taste when Fe is used to fortify foods, which is more noticeable in beverages (Hurrell, 1997).

Many governments in the west have taken the decision to fortify white wheat flour, for example in the UK the Bread and Flour regulations of 1998 make fortification of white flour with Ca, Fe, and thiamine compulsory. While this option is available to countries with a population dependent on purchased and convenience foods, in those countries where a far larger population is more directly dependent on the land, this is neither practicable nor affordable.

Agronomic practices show potential via effective farm management systems to improve soil quality through better fertilization and watering systems. This can positively influence the nutritional status of farm produce. However, systems reliant on water may not be available to regions which often suffer with drought; this is the case in millet consuming communities. There are also a wide range of factors that influence the soil and in turn nutrient uptake, such as light intensity, temperature, and rainfall. These are difficult to control in an agricultural environment; therefore control of nutrient uptake may not be reliable. There are other variables relating to the soil that are difficult to control, such as the proportion of sand, silt, clay, and organic matter, which in turn has a direct effect on mineral composition and uptake by the plant (Hornick, 1992).

Improving Fe and Zn content in staple foods through biofortification is considered to be a cost effective, sustainable and consumer friendly solution in meeting target levels of Fe and Zn in human populations (Velu et al., 2007). The aim of biofortification is to make crops more nutritious as they grow, rather than adding nutrients when processing them into foods. This is largely facilitated by natural cross breeding and genetic improvement methods which rely on natural genetic variation, the use of modern tools for selection and the identification of new genes and gene combinations that can be used to improve levels of Zn and Fe. These techniques can be used to produce elite varieties of pearl millet with increased micronutrient densities.

## Pearl Millet (*Pennisetum glaucum*), an Ideal Candidate for Biofortification

Pearl millet (family: Poaceae, subfamily: Panicoideae) is a multi-purpose cereal crop which provides food, fodder and fuel on more than 27 million hectares worldwide (Jalaja et al., 2016). It has a 2530 Mb genome size and a diploid chromosome number of 7, 2*n* = 14 (Bennett et al., 2000). Pearl millet has evolved under the pressures of infertile soils, heat, and drought, thus giving it a natural ability to thrive in low moisture, nutrient deprived soils and at high temperatures, in excess of 40°C. It is cultivated throughout the arid and semi-arid regions of West Africa, East Africa and many parts of India (Oumar et al., 2008). In these regions pearl millet constitutes up to 75% of the total cereal production, therefore represents an important part of local diets (Lestienne et al., 2005). Pearl millet grains are naturally nutritious when compared to rice and wheat (**Table 2**), and they generally require few chemical inputs; thus, investments in production tend to be low and more suitable for areas that have not benefited from dominant agricultural growth trajectories (Jalaja et al., 2016). It is an example of an orphan crop that is regionally important, but scientific research is still somewhat limited. For example, pearl millet does not yet have a reference genome.

**Table 2:** Nutrient profile comparison of millet with other staples (United States Department of Agriculture Agricultural Research Service, 2016).

Component	Crop (Per 100 g portion, raw grain)			
	Wheat	Rice	Maize	Millet
Energy (KJ)	1368	1537	360	1418
Protein (g)	12.6	7	3	11.3
Fat (g)	1.5	1	1	3.3
Iron (mg)	3.2	1.1	0.2	4.4
Zinc (mg)	2.6	1.1	0.3	1
Calcium (mg)	29	28	2	28
Magnesium (mg)	126	115	89	287

## Biofortified Pearl Millet – Human Trials

The literature reports a large number of studies that have had success from biofortified crops. For example, in a study by Cercamondi et al. (2013) a field trial was conducted on 20 African women from Benin to evaluate the potential of Fe-biofortified pearl millet as a source of additional bioavailable Fe. Results showed that upon consumption of two meals of Fe biofortified pearl millet per day of for 5 days, the total amount of Fe absorbed from Fe-biofortified pearl millet was up to three-times higher than that from regular pearl millet. This suggests that biofortification of pearl millet is a valuable approach in increasing bioavailable Fe to those living in millet consuming communities with limited access to conventional post-harvest fortified foods (Cercamondi et al., 2013). These findings coincide with another recent study conducted in Karnataka, India. In this study by Kodkany et al. (2013) forty Fe deficient children (aged 2 years) were fed pearl millet biofortified with both Zn and Fe. Findings showed that the amount of both Fe and Zn absorbed from the biofortified pearl millet test meals was significantly greater than that from the non-biofortified pearl millet meals and the absorption of both Fe and Zn from the biofortified meals exceeded the minimum physiological requirement for children aged 2 years of 0.54 and 2.5 mg/d, respectively. These findings suggest that increased concentrations of Zn and Fe in pearl millet as a result of biofortification are more than sufficient in meeting the minimum physiological requirements of Zn and Fe in young children and there is vast potential for biofortification in eliminating MNDs, especially within millet consuming communities in developing countries (Kodkany et al., 2013).

## Understanding Iron and Zinc Uptake- From Root to Seed

Any attempt to increase Fe and Zn concentrations in pearl millet grains using traditional breeding methods or genetic engineering must first consider how Fe and Zn are obtained from the environment, distributed and stored (see Morrissey and Guerinot, 2009 for a comprehensive review). Even a small increase in bioavailable nutrient metals in pearl millet grains would have a significant impact on human health, particularly for those living in developing countries.

Initial high levels of nutrient metals from the soil can be toxic. For example, unregulated high affinity binding of Zn to S-, N-, and O-containing functional groups in certain biological molecules and uncontrolled displacement of essential metal cations, for example Mn^2+^ and Fe^2+^, can cause significant damage (Palmgren et al., 2008). In light of this, the activity of metal ion transporters is selective and highly regulated (Philpott, 2014). This is in part achieved through membrane transporters such as metal tolerance proteins (MTPs) (Ricachenevsky et al., 2013). Elucidating the mechanisms behind cation selectivity and regulation is important in understanding plant metabolism and development. If these pathways can be fully understood, they can be improved through biotechnological manipulation. A variety of platforms including, but not limited to, phylogenetic analysis, transcriptomics, gene expression analysis and sequence manipulation are available to help elucidate these mechanisms (see Ricachenevsky et al., 2013 for a comprehensive review).

Fe availability in plants is dictated by a variety of factors including soil redox potential and pH. In soils that are at high pH, Fe is readily oxidized and presents itself as insoluble ferric oxides, however, at lower pH, ferric Fe is released from the oxide, making it available for root uptake via the activity of a ferric chelate reductase, FRO2 (Marschner and Rimmington, 1988). Fe is transported into the root epidermal cells by AtIRT1, a divalent metal transporter which is a member of the ZIP family of transporters. AtIRT1 also transports Zn, Mn, Cn, and Ni (Korshunova et al., 1999). Unknown phenolics then control the extraction of Fe from the negatively charged cell walls, which allows transport into the root symplast. Fe is then bound by unknown chelators or chaperones, and moves symplastically through the connected cytoplasm of the root (Marschner and Rimmington, 1988). At the pericycle, Fe is eﬄuxed into the xylem and moves toward the root via transpiration. When Fe enters the xylem, it complexes with citrate, without which, Fe will not move efficiently through the xylem and won’t be utilized by the shoot (Curie et al., 2009). Fe is then transported into the phloem by nicotianamine (NA) and YSL transporters (Morrissey and Guerinot, 2009). YSLs play an important role in the long distance transport of Fe complexes and seed delivery (Inoue et al., 2009). NA serves as a transporter that facilitates the movement of Fe in and out of the phloem via YSLs (Morrissey and Guerinot, 2009). It also complexes with Fe^2+^ and Fe^3+^ and binds readily to Cu^2+^, Ni^2+^, Co^2+^, Zn^2+^, and Mn^2+^ (Curie et al., 2009). NA also plays an important role in metal homeostasis (Takahashi et al., 2003). Movement within the phloem occurs via ITP (Morrissey and Guerinot, 2009). Fe moves into the seed via the phloem, and seeds in the early stages of development receive Fe from roots and senescent leaves. The loading of Fe into the seed occurs by NA and YSLs and is stored in the endosperm (Morrissey and Guerinot, 2009). A large abundance of Fe may be toxic to the seed embryo, therefore plants possess two damage preventing mechanisms; Fe can either be stored in large plastids with ferritins, which are able to store up to 4500 Fe atoms (Grillet et al., 2014) or Fe can be stored as phytate complexes.

**FIGURE 1:**
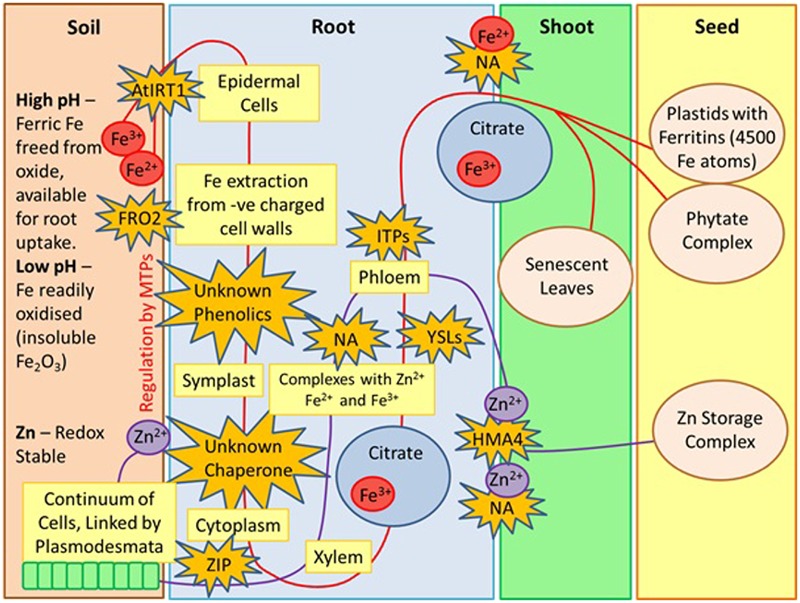
**Zinc and Iron uptake from soil to seed, derived by Author.** MTPs, metal tolerance proteins; FRO2, ferric chelate reductase; ZIP, zinc regulator transporter proteins; AtIRT1, divalent metal transporter; NA, Nicotianamine; YSLs, yellow stripe like transporters; ITPs, Iron transport proteins; HMA4, heavy metal ATPASE 4.

Despite the importance of Zn as an essential micronutrient, there is a significant lack of literature detailing the mechanisms of Zn uptake compared to that of Fe. In the soil, Zn is taken up into the root epidermal cells in its water soluble, +2 oxidation state and unlike Fe^2+^, it is redox stable (Broadley et al., 2007). Several metal transporters of the ZIP family are considered to be the primary uptake systems for Zn (Guerinot, 2000). After uptake, Zn is present in living cells with a neutral pH, therefore it is prone to binding to a wide range of organic molecules. This restricts movement and limits travels between living cells. Zn and Fe are thought to compete for the same uptake systems, therefore NA is also utilized as a transporter (Olsen and Palmgren, 2014). Transport from epidermal cells into the root xylem occurs via a symplasic pathway through a cytoplasmic continuum of cells, which are linked by plasmodesmata. The movement of Zn is then facilitated into the stellar apoplast (Lasat and Kochian, 2000). As discussed previously in the case of Fe, the chelator NA also contributes to long distance transport of Zn from the roots into shoots and seed. NA also modulates the VSC, an essential mechanism controlling the way plant vacuoles provide temporary storage for micronutrients (Sperotto et al., 2014). Zn uses the transported HMA4 in shoot loading and once in the xylem, it is transported in an aqueous form (Olsen and Palmgren, 2014). How Zn enters the phloem is not yet known, however, YSL proteins are likely to play a role in the process. A summary of the above can be seen in **Figure 1**.

There are a variety of factors that affect the efficiency of Fe and Zn uptake including genotype, nutrition, soil type and climatic conditions. For example, plant-associated microorganisms present in the soil can stimulate growth and influence yield and quality of edible parts by affecting nutrient mobilization and transport (Pii et al., 2016). Because of this, the root rhizosphere microbiome, which consists of the area of soil surrounding the root where complex ecological and biological processes occur (Bais et al., 2006), is considered to be one of the key determinants of productivity and plant health. Pii et al. (2016) showed, by using non-metric multidimensional scaling (NMDS) analysis, the presence of the different plant species coupled with nutritional status could promote a differentiation of the rhizosphere microbiome, which in turn has a significant effect on micronutrient uptake. Root exudates are also thought to play a significant role in efficient micronutrient uptake, including that of Fe and Zn. For example, a variety of low and high molecular weight organic compounds and are triggered if plants are exposed to certain abiotic stressors (Dakora and Phillips, 2002) and due to their solubilising, chelating, redox capacities, they play a fundamental role in enhancing nutrient bioavailability, uptake, translocation and allocation. In recent decades it has also been proved that since Fe bioavailability is reduced in aerated and calcareous soils (Kobayashi and Nishizawa, 2012), plants have developed different mechanisms to compensate for Fe shortage including: (i) Retrieving Fe from the soil via the acidification of the rhizosphere through the release of protons, which causes the reduction of Fe^3+^ to Fe^2+^ by FRO2 and (ii) exudation of non-proteinogenic amino acids and uptake with the aid of yellow stripe 1 and yellow stripe 1-like transporters (Kobayashi and Nishizawa, 2012).

## Transcriptomics and Metabolomics Aid a Better Understanding of Zinc/Iron Homeostasis

Understanding gene-to-metabolite pathways by transcriptomics and metabolomics can lead to the identification of gene function and increased production of valuable compounds in pearl millet (Hirai et al., 2004). Although this field is still limited in pearl millet, there have been vast amounts of success with model species. For example, in a study by Assunção et al. (2010), in *Arabidopsis thaliana*, two transcription factors *bZIP19* and *bZIP23* were identified as essential regulators of the response to Zn deficiency. These findings were a significant step in understanding the molecular mechanisms and pathways behind Zn homeostasis, allowing the improvement of Zn biofortification. Findings from the study were also confirmed by Azevedo et al. (2016) and a microarray experiment comparing gene expression between roots of wild-type and the mutant *bzip19 bzip23* (hypersensitive to Zn deficiency), exposed to Zn deficiency, led to the identification of differentially expressed genes concerned with Zn homeostasis. Transcriptome and metabolome data facilitates a better understanding of nutrient metabolism. For example, in a study by Schmid et al. (2014) roots of *A. thaliana* were grown under Fe-replete and two different Fe-deplete conditions and were subject to extensive metabolome analysis by Gas Chromatography Mass Spectrometry (GC-MS) as well as ultra-pressure liquid chromatography electrospray ionization quadrupole time-of-flight mass spectrometry (UPLC-ESI-QTOF-MS). Findings showed that Scopoletin and other coumarins showed a strong response to two different Fe-limited conditions. This suggests that these classes of compounds are released in response to Fe deficiency.

## Biofortification of Pearl Millet (Traditional Plant Breeding and Genetic Modification)

Biofortified pearl millet with elevated Zn and Fe levels may be achieved either through conventional plant breeding methods or through the use of transgenic techniques (Bouis and Welch, 2010). Biofortification is largely facilitated by drilling down into the genomes of crops to seek genes of interest and to breed these genes into new, improved varieties. In a society where agricultural success is highly focused on calories, plant breeders often focus on 1000 grain weight. As a result of this, nutritional value is often ignored. The end-product should therefore be sustainable by increasing nutrition, whilst keeping in mind the importance of a high 1000-grain weight.

Despite the lack of a reference genome, genetic improvement of pearl millet results from the use of available genetic resources, which facilitates the selection and breeding of elite cultivars with high nutritional value. Genetic and genomic technologies drive the way forward for the discovery and transfer of genes and QTL associated with an improved nutritional profile from the diverse genetic resources of millets (Muthamilarasan et al., 2016). A pearl millet sequencing consortium^1^ are also in the process of developing a reference genome, and it is expected that findings will be publically available soon.

## Germplasm Collections – A Good Place to Start

Managed germplasm collections are available for pearl millet, and characterisation of genetic diversity within these collections is a necessary prelude to their efficient use (Varshney et al., 2009). A wide variety of pearl millet germplasm collections exist on a global scale, including the Pearl Millet inbred Germplasm Association Panel (PMiGAP) developed at International Crops Research Institute for the Semi-Arid Tropics (ICRISAT), Patancheru, India. The PMiGAP has been drawn from a core collection of 1000 pearl millet accessions, landraces and cultivars grown across three continents (Sehgal et al., 2015). ICRISAT has also developed other collections including the Iniari germplasm, which uses landraces from West Africa, which have superior grain filling abilities under terminal drought stress, larger seeds, thicker panicles, and broader leaves (Ito et al., 1999). Other collections include The USDA National Plant Germplasm System Pearl Millet Collection, which is maintained at the Plant Genetic Resources Conservation Unit located in Griffin, Ga, USA. It contains 1297 unique genotypes from 31 countries^2^.

## Traditional Breeding Methods

Traditional breeding methods involve the selection of two parental lines with high Fe and Zn content and crossing them to create a hybrid that expresses the traits of interest. Successful crop improvement via plant breeding largely depends on the existence of genetic variation for the target traits within the gene pool. When breeding for elevated levels of Fe and Zn in edible parts, the task is further complicated by the fact that the grain micronutrient concentration highly depends on environmental conditions, including soil mineral composition (Feil et al., 2005). GEI are therefore a major factor in the development of stable and high-nutrition cultivars of pearl millet (Moghaddam and Pourdad, 2009) and must be accounted for. In light of this, multi-environmental trials are required to verify the stability of phenotypic data. The presence GEI may reduce the validity of any analysis, restrict the significance of findings, and limit the efficiency of selecting elite genotypes (Gurmu et al., 2009).

## A Stable Phenotype and Maintaining Yield

Many pearl millet studies have identified potential high Fe and Zn lines with stable phenotypes, including one by Velu et al. (2007) who analyzed a diverse range of genetic materials developed at ICRISAT for grain Fe and Zn content. Based on the average performance in two growing seasons, large genetic variability among the entries was found for both Fe and Zn. Well-adapted, highly utilized genotypes and their progenies from the Iniari germplasm contained high levels of grain Fe and Zn density and large within-population genetic variability for Fe and Zn was reported. The correlation between Fe and Zn content was found to be positive and highly significant. This suggests that the simultaneous selection for elevated levels of both micronutrients is possible, and selection within the Iniari germplasm is likely to provide excellent candidates for the development of elite varieties with increased grain Fe and Zn content (Velu et al., 2007).

When considering traditional methods for biofortification for reducing MNDs, it is important to consider sustainability coupled with socioeconomic factors for smallholder farmers – the people that will directly benefit from pearl millet research. For example, genes associated with micronutrient uptake and their relationship with grain yield has a direct bearing when formulating effective strategies for breeding elite lines (Kanatti et al., 2014). In terms of increasing micronutrient content and keeping high yield, the study by Velu et al. (2007) also showed highly significant positive correlations of 1000-grain weight with Fe and Zn content per grain indicated that breeding for elevated levels of these micronutrients is possible without compromising yield.

Kanatti et al. (2014) demonstrates, using 196 hybrids and their 28 parental lines of pearl millet, that in order to breed successful hybrids that express elevated grain Fe and Zn levels, the same genes for Fe and Zn content should be incorporated into both parents (Velu et al., 2011). Hybrids were found to express no better-parent heterosis as barely any hybrid was found transgressing the parental lines for increased grain Fe content. The study showed that the underlying physiological processes that determine grain Fe and Zn content are primarily under additive genetic control (Govindaraj et al., 2013; Kanatti et al., 2014) and a large amount of the mid-parent heterosis values were in the negative direction. This indicates that the involvement of genes, with the exception of those with additive gene action, where alleles determining lower Fe and Zn densities, are partially dominant. However, when considering additive gene action, if the same source is used to transfer the genes associated with Fe and Zn content in both parental lines, this would cause amount of genetic diversity between lines for other traits to be reduced. This may lead to a reduction in heterosis for yield, which is controlled by non-additive gene effects (Kanatti et al., 2014). It was also found that Fe and Zn content from inbred lines and their general combining ability were positively correlated and highly significant. This suggests that recurrent selection could be used to significantly improve breeding populations for grain Fe and Zn content and breeding lines selected for high Fe and Zn levels are more likely to include those with high general combining ability for these micronutrients (Govindaraj et al., 2013).

## End Use Quality

End-use quality is an important factor to consider when improving any grain crop quality trait. The acceptability of a cultivar by farmers and consumers is highly based on how the grain is processed and end-use quality (Ortiz-Monasterio et al., 2007). Additionally, the concentration and bioavailability of micronutrients in pearl millet, as in other cereal crops, may be enhanced or reduced by various methods of processing, this is achieved by fortification with certain ingredients and meal preparation techniques (Welch and Graham, 2004). Therefore, end-use quality traits including protein content, grain hardness and baking properties must be considered when creating elite lines in pearl millet. For example, in maize, grain hardness and factors affecting the gelatinisation and pasting properties of starch (Ramirez-Wong et al., 1994) are considered when determining end-use quality. Micronutrient-enhanced lines should also be screened for desirable end-use quality traits. For example, various processing treatments of pearl millet including germination, autoclaving, and debranning are known to be effective in reducing levels of phytate (Sharma and Kapoor, 1996). However, some studies suggest that some methods of processing raw pearl millet grain may result in decreased levels of Fe. For example, soaking of grains results in a 25% loss of Fe (Eyzaguirre et al., 2006). In light of this, methods of processing should be considered that are not detrimental to the levels of these nutrients.

## Toxic Effects

Any potential products of biofortification should be carefully evaluated under real conditions. This can be achieved via the assessment of trace element bioavailability to humans and investigation into any drawbacks such as enhanced uptake of toxic metals (e.g., Cd, which is deleterious to all organisms). Toxic metals such as Cd share the same transporters as some micronutrient metals (e.g., Fe and Zn) (Zhao and McGrath, 2009). Several studies have investigated of the possible unwanted side effects of biofortification, for example via the enhancement of ZIPs. In addition to aiding Zn uptake by roots, ZIPs can also aid the uptake for other, non-specific cations such as Cd. Two well-characterized ZIP family proteins, *IRT1* and *IRT2*, represent the main Fe^2+^ uptake systems in *A. thaliana* root cells. *IRT1* also facilitates the uptake of Zn and Cd (Guerinot, 2000). If this pathway was enhanced by biofortification, this could cause the plant to become toxic and dangerous for consumption.

## The Effect of Phytate on Mineral Bioavailability

The improvement of Fe and Zn levels in plants would also need to include the reduction of antinutrient compounds such as phytate. Due to high phytate content within the endosperm of pearl millet seed, this often limits the bioavailability of many important micronutrients such as Fe and Zn upon human consumption (Shanmuganathan et al., 2006). The hindering effect of phytate on mineral bioavailability has been confirmed by Egli et al. (2004) whose *in vivo* radioactive and stable isotope studies demonstrate that, absorption of Fe, Zn, and Ca are significantly lower from diets with a high content of phytate than from diets that contain low levels of phytate.

**FIGURE 2:**
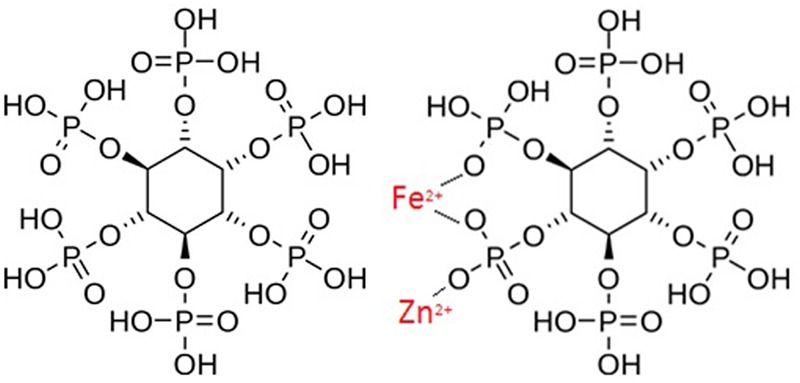
**Left: Chemical Structure of Phytate.** Bottom: How phytate chelates with iron and zinc, derived by Author.

Phytate exists as a phosphorylated myo-inositol ring which strongly chelates metal cations, including Fe^2+^ and Zn^2+^ (**Figure 2**) (Urbano et al., 2000). When Fe and Zn bind to phytate, an insoluble precipitate is formed. This is not efficiently absorbed by the intestines due to the absence of intestinal phytase enzymes (Nielsen et al., 2013). This poor absorption can therefore exacerbate Fe and Zn deficiencies (Hurrell, 2003). The adverse effect of phytate on Fe and Zn absorption is dose-dependent (Gibson et al., 2010). For example, studies have proved a negative relationship between Zn absorption and phytate over a wide range of phytate: Zn molar ratios (Hambidge et al., 2004). With respect to Fe, the inhibitory effect of phytic acid is still strong at very low phytate levels, when ratios are as low as 0.2 Phytate: 1.0 Fe (Hallberg et al., 1989).

Although indigestible to humans, phytic acid plays several key roles in the development of seedlings. For example, phytic acid acts as the principal storage form of P and also acts as a source myoinositol, which is required for cell wall development (Reddy et al., 1982). Because of this, phytate can never be completely eliminated from the crop. However, conscious efforts should be made to significantly reduce phytate so that it does not become a limiting factor in micronutrient absorption. A possible solution is the development of low phytate varieties of pearl millet. For example, in 2006 a study by Shanmuganathan et al. (2006) showed that many parents and crosses can be successfully exploited for the development of pearl millet genotypes with low phytate content. The study involved crossing 11 pearl millet genotypes to create 55 hybrids and measuring the hybrids for phytate content in order to understand the nature of gene action and to evaluate the parents and hybrids for combining ability with respect to low phytate content. Several crosses were identified as having low phytate content by measuring negative specific combining ability.

Even though a large number of pearl millet lines have been associated with low phytate content, for example in a study conducted by Simwemba et al. (1984) two lines (TIFT 2 23 DAE X 656 + 653) were identified to have low phytate content than that of certain varieties of wheat, it was also found that the environment (as well as genetics) also appears to play an important role in determining phytate content (Simwemba et al., 1984). There also appears to be a significant gap in the literature as no genes/markers have been discovered with an association with low phytate content in pearl millet. It is hoped that using synteny studies with other crops may facilitate the discovery of markers/QTLs/genes associated with low phytate content (Rawat et al., 2013). For example, QTL mapping for phytate content has been accomplished in rice (Stangoulis et al., 2007), soy bean (Walker et al., 2006), and bean (Cichy et al., 2009). It has also been proved that loci affecting phytate content are different than loci affecting grain micronutrient content, this suggests that the simultaneous increase of grain micronutrient content and a decrease in phytate content is possible (White and Broadley, 2011). For example, in the case of Stangoulis et al.’s (2007) rice study, QTLs for grain phytate, Zn and Fe concentration were identified in several rice lines from an IR64 × Azucena doubled haploid population. Findings showed that there were significant positive correlations between phytate levels and Fe/Zn concentrations. Since the QTLs of phytate are located on different chromosomal regions compared to those found for Fe and Zn, this suggests that they are genetically distinct and it should be possible to use molecular markers for breeding and selection purposes to modify the phytate concentration without affecting grain micronutrient content (Stangoulis et al., 2007).

Following these studies, further population improvement should be implemented using recurrent selection to breed for low phytate content, whilst keeping yield and micronutrient uptake high.

## Tools that Harness the Potential of Pearl Millet in the Field of Genetics and Genomics

In recent decades, the potential of pearl millet and genetic variations associated with beneficial phenomic traits has been recognized. However, the use of molecular breeding technology for the genetic improvement of pearl millet is somewhat limited and progress is slow due to insufficient numbers of PCR compatible co-dominant markers (Senthilvel et al., 2008).

## Genetic Maps

Genetic maps enable a phenotypic trait to be linked to a gene or a region on a chromosome. Previously, genetic maps were highly dependent on morphological markers. However, recent advances in biotechnology have facilitated the creation of high-density maps that consist of thousands of molecular markers (Hyten and Lee, 2016). Recently, the use of an F2 population of 93 progenies and 9 cultivated pearl millet crosses has facilitated the production of a genetic map with higher density and better uniformity of markers than previously published maps. This was achieved using a modified GBS platform, which involved two restriction enzymes (*PstI–MspI*) and PCR amplification with primers including three selective bases. These efforts resulted in 3,321 SNPs generated for public use (Moumouni et al., 2015). The availability of large numbers of SNP markers and high-density genetic maps will enhance the progress of gene and QTL mapping in biparental populations significantly, and facilitate association analyses on panels of unrelated lines.

## Genome Wide Association Studies

Phenotypic data is combined with GBS data in order to identify genomic regions controlling traits of interest via GWAS. GWAS have expanded into a powerful tool for investigating the genetic architecture in many staple crops. GWAS exploits the natural diversity generated by multi-generational recombination events that occur in a population or germplasm panels (Deschamps et al., 2012). This approach results in increased mapping resolution as compared to linkage mapping populations. Genetic sources of phenotypic variation are an essential component of plant genetics. Taking on the lessons learned from model species, such as rice and maize, future developments are being applied to staple crops as well as orphan crops. Comprehensive maps of genome variations will facilitate GWAS of complex agriculture benefitting traits in crops. The result of which will greatly accelerate the improvement of crops via genomics-assisted breeding (Huang and Han, 2014). Recent advances toward whole genome sequencing of the pearl millet genome together with resequencing of the entries of the various germplasm populations will certainly assist in such endeavors. Findings from GWAS will be the catalyst in the mining of candidate genes. These candidate genes can be verified through Transfer (T)-DNA mutants or genetic transformation, which will then facilitate the genetic modification or MAS for validated genes. These steps will then lead to more nutrient rich, improved varieties (Huang and Han, 2014).

## Synteny Studies and Resources From Major Crop Species

Synteny studies among the grass family sets the stage for a comparative study of millets and non-millet cereals to trace common genes associated with nutrition biosynthesis pathways (Muthamilarasan et al., 2016). When these genes, alleles and QTLs are discovered, they can then be incorporated into elite lines through the use of molecular marker assisted breeding or transgene based methods. Synteny studies may also facilitate the introgression of these traits of interest from the major cereals into millets. This will largely be achieved with the role of genomics, bioinformatics, transcriptomics, proteomics, metabolomics, and ionomics (Muthamilarasan et al., 2016). The dearth of genomic resources that has characterized most major cereal crops is going to benefit pearl millet directly. The high throughput and low cost of NGS technologies has made it possible to sequence crops with lower economic value for the development of elite cultivars with desirable traits. Because significant genomic collinearity has been reported in many cereal crops (Devos, 2005), comparative genomics methods that are facilitated by the use of genomic resources and bioinformatics tools will allow the transfer of genes from model/major crops to minor crops (Varshney et al., 2006). The benefits of this include (i) improved analysis of cereal biodiversity and the identification of useful variants; (ii) MAS of alleles and allele combinations of interest; and (iii) cloning and efficient transfer of useful alleles among members of the cereal family (Nelson et al., 2004). Resources from well sequenced species will enable functional definition of many key genes and pathways (Chen et al., 2016). For example, Chen et al. (2016) studied the metabolic and pGWAS (mGWAS and pGWAS) in rice grain and maize kernels and identified new candidate genes that could be the cause of variation in traits such as grain color and size. It was found that distinct and overlapping aspects of genetic control of metabolism exist within and between species and the mGWAS analysis indicated that the rice and maize are likely to share common genetic control strategies for a variety of metabolites. A search for homologous loci mapped by the same metabolites (or metabolites with similar structures) identified 42 loci underlying the 23 co-detected metabolic features between maize and rice. This data suggests that there is potential for the identification of genes associated with traits of interest between cereals using mGWAS and pGWAS and genetic analysis of the metabolome could improve what is currently known about these complex traits.

## QTL Fine Mapping

Quantitative trait loci associated with increased Zn and Fe accumulation in pearl millet are an important asset to those targeting candidate genes associated with these traits and continue to drive biofortification research. Focus should be made on what resources could provide potential candidates for the identification of QTLs. As discussed, there are several germplasm panels that hold lines associated with high micronutrient accumulation that cover global diversity. Potential candidates for QTL fine mapping exist within these germplasm banks. For example, in the Iniadi pearl millet germplasm, certain lines have been found to be particularly high in Fe and Zn, with a highly significant and high positive correlation between these two micronutrients. One of these lines, ICTP 8203 has been released in India and in 1995 it was cultivated on more than 0.8 million hectares (Rai et al., 2013). At present, ICTP 8203 is still cultivated on about 0.2 million hectares, predominantly within Maharashtra, India (Bantilan and Joshi, 1998). Simultaneous accumulation has been reported in a wide variety of crops, including pearl millet and these positive correlations could be due to common and overlapping QTLs for grain Fe and Zn densities (Kumar, 2011).

Research into identifying QTLs and candidate genes for elevated levels of Fe and Zn in pearl millet is limited at this time, perhaps due to resource constraints such as lack of a reference genome. The fact that QTLs associated with the trait have been identified in other crops will benefit QTL fine mapping in pearl millet through synteny studies. For example, QTLs for grain Fe and Zn densities as reported in wheat (Peleg et al., 2009), rice (Stangoulis et al., 2007), and bean (Cichy et al., 2009). For example, in a tetraploid wheat population of 152 RILs, 82 QTLs were mapped for 10 minerals, including Fe and Zn with LOD score range of 3.2–16.7. These were located in 32 non-overlapping genomic regions. A strong association was found between QTLs conferring Zn and QTLs for Fe, which is indicative of a strong genetic association between mechanisms affecting grain Zn and Fe levels (Peleg et al., 2009).

## Recombinant DNA Technology

Recombinant DNA technology is a powerful tool that facilitates the improvement of the pearl millet gene pool (O’Kennedy et al., 2006). Research into DNA technology has been developed extensively in major cereal crops, more so than for pearl millet. Although recent advances for the improvement of pearl millet have been well established via traditional breeding methods and MAS, genetic engineering and *in vitro* culture allows the gene pool to be expanded further than previously thought possible. This is facilitated by the transfer of genes which control well-defined traits between species (O’Kennedy et al., 2006).

The first cereal embryogenic *in vitro* culture systems were established for pearl millet in the 1980’s (Vasil and Vasil, 1981) and since then, efficient regeneration systems for pearl millet breeding lines have facilitated the development of reliable transformation systems (O’Kennedy et al., 2006). *In vitro* culture and transformation of pearl millet is established and reliable protocols have been developed and can be further improved thanks to the progress of transformation in major crop species (Oldach et al., 2001; Goldman et al., 2003). For example, improved nutritional quality and better genetic engineering methods can be used to elevate levels of minerals and vitamins in the starchy endosperm of cereal seeds. This has been accomplished in rice, where expression of soybean ferritin (a Fe binding protein) in developing seeds of rice has resulted in a threefold increase in endosperm Fe content compared to the non-transformant (Qu et al., 2005). This work was achieved using two types of ferritin hyper-expressing rice lines, which were synthesized via the introduction of a soybean ferritin *SoyferH-1* gene under the control of the rice seed storage glutelin gene promoter, *GluB-1* and the rice seed storage globulin gene promoter, *Glb-1*, (Double transformation line *GluB-1/SoyferH-1* and *Glb-1/SoyferH-1*) and by introducing the *SoyferH-1* gene under the control of *Glb-1* promoter alone (Single transformation line with *Glb-1/SoyferH-1*). Similar findings were also reported by Goto et al. (1999) where the coding sequence of the soybean ferritin gene was transferred into Asian rice by Agrobacterium-mediated transformation. *GluB-1* was used to facilitate the expression of the soybean gene in developing, self-pollinated seeds of transgenic plants. Findings showed that the Fe content of seeds from the transgenic plants was up to three times greater than that of their untransformed counterparts (Goto et al., 1999). The same techniques could be applied to pearl millet for the increased Fe accumulation.

Harnessing tools that facilitate genetic engineering have also been established for anti-nutrient compounds such as phytate. The pathway of phytate from myoinositol is also considered to be well understood and the screening of mutant populations for reduced phytate accumulation is now possible. For example the identification of low phytate mutants in maize, barley, wheat, soybean, and rice will assist in the selection of similar mutations millets and incorporated into breeding programs. In barley, grains were mutagenised with sodium azide and screened for high levels of free phosphate for the identification of low-phytate mutants. Results showed that nine out of 27 mutants had an increased free phosphate content in the grain and this was correlated with a significant decrease in levels of phytate. Allelic testing of four out of the nine mutants showed that at least two distinct loci control the biosynthesis of grain phytin (a calcium magnesium salt of phytic acid). It is therefore possible to screen for and isolate low phytate mutants through identification of genes involved in the biosynthetic pathway of phytin. This contributes to the development of low-phytin crops with higher nutritional value (Rasmussen and Hatzack, 1998).

As previously discussed, NA, plays a key role in metal assimilation and homeostasis (Morrissey and Guerinot, 2009). Therefore, manipulation of cellular NA concentrations should be considered for the improvement of Fe and Zn content pearl millet. This has been achieved through the use of activation and knockout mutants in rice and tobacco. A study by Inoue et al. (2003) demonstrates that among the three *OsNAS* genes present in rice, *OsNAS1* and *OsNAS2* transcripts are elevated in roots and leaves in response to reduced Fe levels, whereas *OsNAS3* expression is induced in roots but suppressed in leaves when Fe is insufficient. Activation and knockout mutants were used to examine the functioning of *OsNAS3* in metal homeostasis in rice plants and it was found that there was an increase in NA by activation of *OsNAS3*, causing increased levels of Fe and Zn in both leaves and seeds (Lee et al., 2009). *NAS* genes could therefore be potential candidates for the improvement of Fe and Zn in rice. Constitutive overexpression of *NAS* genes also resulted in elevated levels of Fe and Zn in transgenic tobacco plants (Douchkov et al., 2005), this suggests findings may also be relevant to other crops such as pearl millet through synteny studies.

## CRISPR Gene Editing

The development of Clustered Regularly Interspaced Short Palindromic Repeats (CRISPR) relies on the enzyme *Cas9* that uses a guide RNA molecule to target specific DNA sequences, and then edits the target DNA to either disrupt genes, leading to genome modifications during the repair process or to insert new sequences (Ledford, 2015). This method is desirable in the field of crop science because it is highly efficient, robust, is associated with reduced risk and enables a wide variety of agricultural applications. Genetic transmission of edits has been reported in *A. thaliana*, rice, tomato, and sorghum (Jiang et al., 2013; Zhou et al., 2014). In Rice, large chromosomal segment deletions, the inheritance of genome mutations in multiple generations and the construction of a set of facile vectors for highly efficient, multiplex gene targeting has been reported. In a study by Zhou et al. (2014) four sugar eﬄux transporter genes were successfully modified at high efficiency. The most efficient system yielded 87–100% editing at the target sites in T0 transgenic plants with the rice codon optimized *Cas9*, all with di-allelic mutations. Genetic crosses segregating Cas9/sgRNA transgenes away from edited genes yielded several genome-edited but transgene-free rice plants (Zhou et al., 2014). These established methods and protocols could benefit pearl millet as the results of these studies suggest evidence that the Cas9/sgRNA systems are fully functional, reliable and efficient in two model plant and two major crop species and suggest this system has vast potential tool for manipulation of plant genetics.

Due to the orphan status of pearl millet, little work has been performed so far on the nutritional enhancement of their grains via genetic engineering – thus presenting a significant gap in the literature. However, work on major cereals via reliable techniques and protocols have demonstrated that genetic improvements are possible; using genetic engineering approaches (O’Kennedy et al., 2006). In order to employ recombinant DNA technology methods, there needs to be an increase in knowledge about the Zn and Fe pathways in pearl millet and it is also important to consider factors such as cost, consumer acceptability and socioeconomics to small holder farmers.

## Future Challenges

The current state biofortification research in pearl millet for nutritional purposes is still limited at this time, as compared to major crop species (Shivran, 2016). In light of this, combined efforts are needed from all fields relating to agriculture, medicine, nutrition and genetics to drive this research in a safe and consumer friendly way (Rahal and Shivay, 2016). To facilitate this research, Fe and Zn pathways in pearl millet must be better understood. A better understanding would increase the safety of transgenic techniques and biotechnological applications, thus making the end-product more reliable and consumer friendly. The mechanisms behind efficient Fe and Zn uptake for improved health and productivity could be better understood by research into the root rhizosphere, dissecting the complex biological and ecological processes within the soil microbiome, elucidating the mechanisms behind cation selectivity and investigation into potential downsides such as enhanced accumulation of antinutrients and toxic metals. Since pearl millet is commonly grown in infertile soils, which are lacking in Fe and Zn, any potential technologies should also be evaluated under real conditions. This could be greatly facilitated by transcriptomics and metabolomics. As demonstrated, the molecular mechanisms of Zn and Fe homeostasis in nutrient rich and nutrient lacking soils have been researched extensively in *A. thaliana* using transcriptomics and metabolomics (Schmidt et al., 2014; Azevedo et al., 2016). The well-established theories and protocols from model species could be applied specifically to pearl millet, allowing further improvement and increased efficiency of Zn and Fe biofortification.

Biofortified pearl millet with increased Zn and Fe content may be achieved either through conventional plant breeding methods or through the use of transgenic techniques. Genetic improvement of pearl millet results from the use of available genetic and bioinformatic resources coupled with extensive phenotyping of diverse germplasm collections. The usefulness of the available germplasm collections is not disputed, although extensive GEI analysis is needed for accurate predictions of how genotypes will suit different environments for efficient nutrient uptake, this can be achieved through real time multi environmental trials to verify the stability of phenotypic data.

Traditional breeding methods highly depend on the existence of genetic variation for the target traits in the gene pool. However, whilst there are a number of studies that show this for Fe and Zn, there are a variety of other complex factors to a consider such as; maintaining high yield and good end use quality, which can be affected by a variety of factors such as levels of phytate and certain methods of processing. In light of this, biofortification should encompass a wide range of factors and not be solely focused on increasing Fe and Zn content.

Advances in DNA-based molecular markers have already contributed to the identification and tagging of some agronomically important genes and QTLs for agricultural applications. The availability of large-scale genome-wide markers in pearl millet will further improve the tagging of nutrition relevant genes and QTLs in pearl millet, and it is hoped that as the awareness of capturing genetic variations for micronutrients in the genome increases, this will facilitate the discovery and validation of genes associated with high micronutrient uptake (Kumar et al., 2016; Muthamilarasan et al., 2016) and lead to improved varieties that can be accessed by small holders and the wider population.

Even though nutritional enhancement of pearl millet grains via genetic engineering should be considered as an important area of research for nutrition security, little work has been performed on pearl millet so far. However, work on model species and major cereals have demonstrated that genetic engineering methods are possible through the use of *in vitro* culture systems, activation and knockout mutants and gene editing methods such as CRISPR (O’Kennedy et al., 2006). This research should be employed when the Zn and Fe pathways in pearl millet are better understood whilst considering socioeconomic factors such as consumer acceptability and feasibility to small holder farmers.

## Conclusion

It is well documented that there are severe health limiting consequences as a result of Fe and Zn deficiencies. Prevention of these deficiencies is therefore perceived as a desirable worldwide goal. The tragic loss of human potential predominantly applies to people living in poverty stricken areas, who depend on grain from staple crops that they eat on a daily basis. The lack of nutritious food has forced many people to depend on food fortification, supplements and agronomic practices as interventions. However, these are not sustainable and suffer major drawbacks. Biofortification of staple crops, such as pearl millet, is considered to be the most sustainable method of intervention and is largely facilitated by traditional plant breeding and transgenic techniques.

Due to the potential role of pearl millet in addressing the challenges associated with MNDs, efforts are being made to target genes responsible to efficient Zn and Fe uptake to be bred into elite varieties. This will have a snowball effect as well-nourished children grow up to be stronger adults. Any attempt to increase Fe and Zn levels via traditional breeding or genetic engineering must first consider the mechanisms behind Fe and Zn uptake, distribution and storage. Genetics and functional genomic platforms have driven research that enhances the productivity, sustainability and nutritional quality of food production systems for a number of years (Asins, 2002) and it is now possible identify QTLs and candidate genes that warrant further investigation to determine if these are accountable for beneficial traits, despite the lack of a reference genome.

The use of available genetic resources and diverse germplasm collections will facilitate this research. A positive and highly significant correlation between Fe and Zn has been demonstrated extensively, therefore there are good prospects for increasing levels of both micronutrients simultaneously, which can be achieved without compromising yield (Velu et al., 2007). It has also been reported that pearl millet lines express ‘no better parent heterosis’ which suggests that the same genes for increased Fe and Zn density should be acquired from both parents (Kanatti et al., 2014) when employing traditional breeding methods. The treatment of raw grains have also been assessed for keeping end use quality high, for example debranning decreases levels of phytate but soaking accounts for up to 25% Fe loss. Therefore, methods of processing should be considered to suitably benefit those suffering from Fe and Zn deficiencies (Eyzaguirre et al., 2006). The potentially toxic side-effects of increasing levels of Fe and Zn have also been evaluated. For example, the simultaneous uptake of Zn and Cd should be accounted for and levels antinutrient compounds such as phytate should be reduced. In light of this efforts have been made to develop low phytate lines in pearl millet.

There are a variety of platforms that aid the development of nutrient rich pearl millet, including the use of genetic maps, GWAS, synteny studies, QTL fine mapping for targeting candidate genes and genetic engineering technology. Using existing genomic resources from other important crops and synteny among the cereal family, common genes associated with nutrition biosynthesis pathways can be identified among millets and non-millets and the introgression of these pathways can be incorporated into pearl millet through either transgene based techniques or traditional breeding methods. Although transgene technologies have been established in many major crop species, methods of gene editing (CRISPR), *in vitro* culture systems, activation and knockout mutants are still being developed in pearl millet.

## Author Contributions

HM: Author of the manuscript, wrote and designed manuscript, implemented and revised during this process. RY and HB: participated in revising it critically for important intellectual content.

## Conflict of Interest Statement

The authors declare that the research was conducted in the absence of any commercial or financial relationships that could be construed as a potential conflict of interest.

## Footnotes

1 http://ceg.icrisat.org/ipmgsc/

2 https://scisoc.confex.com/scisoc/2015am/webprogram/Paper95640.html

## Abbreviations

AtIRT1: iron regulated transporter 1
Fe: iron
FRO2: ferric reduction oxidase 2
GBS: genotyping by sequencing
GEI: genotype × environment interactions
GWAS: genome wide association studies
HMA4: heavy metal ATPase 4
ITP: iron transport proteins
LOD: logarithm of the odds (to the base 10)
MAS: marker assisted selection
mGWAS: metabolomics Genome Wide Association Studies
MNDs: micronutrient deficiency disorders
NA: nicotianamine
NGS: next generation sequencing
PCR: polymerase chain reaction
pGWAS: phenotypic Genome Wide Association Studies
QTL: quantitative trait loci
Redox: reduction-oxidase reaction
RILs: recombinant inbred lines
SNP: single nucleotide polymorphism
VSC: vacuole sequestration capacity
YSL: yellow stripe like transporter
ZIP: Zrt/Irt like proteins
Zn: Zinc
